# Enhancing synchrotron radiation micro-CT images using deep learning: an application of Noise2Inverse on bone imaging

**DOI:** 10.1107/S1600577525001833

**Published:** 2025-04-01

**Authors:** Yoshihiro Obata, Dilworth Y. Parkinson, Daniël M. Pelt, Claire Acevedo

**Affiliations:** a Department of Mechanical and Aerospace Engineering University of California San Diego San Diego CA92161 USA; b Advanced Light Source Lawrence Berkeley National Laboratory Berkeley CA94720 USA; c Leiden Institute of Advanced Computer Science (LIACS) Universiteit Leiden 2333 CALeiden The Netherlands; University of Malaga, Spain

**Keywords:** microtomography, convolutional neural networks, machine learning, bone imaging, Noise2Inverse

## Abstract

In bone-imaging research, *in situ* synchrotron radiation micro-computed tomography (SRµCT) mechanical tests are used to investigate the mechanical properties of bone in relation to its microstructure. Low-dose computed tomography (CT) is used to preserve bone’s mechanical properties from radiation damage, though it increases noise. To reduce this noise, the self-supervised deep learning method Noise2Inverse was used on low-dose SRµCT images where segmentation using traditional thresholding techniques was not possible. Simulated-dose datasets were created by sampling projection data at full, one-half, one-third, one-fourth and one-sixth frequencies of an *in situ* SRµCT mechanical test. After convolutional neural networks were trained, Noise2Inverse performance on all dose simulations was assessed visually and by analyzing bone microstructural features. Visually, high image quality was recovered for each simulated dose. Lacunae volume, lacunae aspect ratio and mineralization distributions shifted slightly in full, one-half and one-third dose network results, but were distorted in one-fourth and one-sixth dose network results. Following this, new models were trained using a larger dataset to determine differences between full dose and one-third dose simulations. Significant changes were found for all parameters of bone microstructure, indicating that a separate validation scan may be necessary to apply this technique for microstructure quantification. Noise present during data acquisition from the testing setup was determined to be the primary source of concern for Noise2Inverse viability. While these limitations exist, incorporating dose calculations and optimal imaging parameters enables self-supervised deep learning methods such as Noise2Inverse to be integrated into existing experiments to decrease radiation dose.

## Abstract 2

The use of Noise2Inverse in low-dose *in situ* synchrotron micro-computed tomography experiments shows high feasibility and promise for biological tissues such as bone. With appropriate scanning parameters and experimental setup, features in bone such as lacunae volume and shape as well as mineralization can be reasonably preserved while reducing radiation dose by two to three times.

## 1. Introduction

In mechanical engineering, materials science and biomedical research, micro-computed tomography (µCT) is widely used to provide 3D images of a material’s internal structure at the microscale (Schladitz, 2011 ▸; Ritman, 2011 ▸). In particular, µCT is extremely relevant in the realm of biological materials, such as bone, due to their characteristically complex hierarchical microstructures (Obata *et al.*, 2020 ▸). Synchrotron radiation micro-computed tomography (SRµCT) takes advantage of high-flux X-ray beams available at synchrotron facilities to image at high speed, high resolution and in 3D at the microscale (Salomé *et al.*, 1999 ▸). With short acquisition time and high resolution, SRµCT is well suited for *in situ* mechanical testing under temperature, pressure or loading to determine how material microstructure evolves under realistic failure conditions (Barnard *et al.*, 2016 ▸). SRµCT imaging of the evolution of a material over time is referred to as 4D SRµCT and is used to investigate spatial microstructural changes as a function of a fourth dimension – time (Voltolini *et al.*, 2017 ▸). In bone, SRµCT has been shown to successfully resolve internal microstructural changes associated with aging and diseases (Peyrin, 2009 ▸; Zimmermann *et al.*, 2011 ▸; Woolley *et al.*, 2023 ▸), and possesses high potential to investigate the evolution of failure mechanisms through *in situ* SRµCT mechanical testing (Peña Fernández *et al.*, 2021 ▸; Claro *et al.*, 2023 ▸; Madi *et al.*, 2020 ▸).

While *in situ* SRµCT mechanical testing enables imaging of microstructural features during damage evolution, this comes at the cost of repeated X-ray exposures, and thus increased radiation dose (Peter & Peyrin, 2011 ▸). The challenge in biological tissues (*i.e.* bone) is that exposure to high levels of synchrotron radiation deteriorates collagen’s natural mechanical properties (Sauer *et al.*, 2022 ▸; Barth *et al.*, 2011 ▸). As a consequence, there is a need to limit scanning time and exposure time for biological tissues and materials prone to radiation damage. Both exposure time and the number of images taken during an SRµCT scan can be reduced to lower the total radiation dose. Both factors tend to reduce image quality and increase noise (Bayat *et al.*, 2005 ▸); thus, an approach for improving SRµCT image quality through denoising must be pursued to expand the feasibility of *in situ* SRµCT testing to biological tissues.

Machine learning has emerged as the state-of-the-art approach for denoising images (Kaur *et al.*, 2018 ▸; Thakur *et al.*, 2021 ▸). Specifically, deep learning methods using convolutional neural networks (CNNs) have had success in complex tasks such as denoising and segmenting SRµCT images (Zhang *et al.*, 2022 ▸). Combining low-dose imaging and deep learning methods for denoising and segmentation may greatly enhance the feasibility of *in situ* SRµCT mechanical testing for biological tissues. Of the many CNN methods for denoising SRµCT images, both supervised and unsupervised denoising techniques have garnered interest in recent years (Yu *et al.*, 2023 ▸; Liu *et al.*, 2020 ▸; Bazrafkan *et al.*, 2021 ▸; Meng *et al.*, 2020 ▸; Kim *et al.*, 2020 ▸). Supervised learning involves the use of labeled training data, in this case a high-quality reference scan, for a denoising CNN to use as a target (Rajoub, 2020 ▸; Muller *et al.*, 2023 ▸). In contrast, unsupervised learning does not require labeled data, *i.e.* a high-quality reference scan (Usama *et al.*, 2019 ▸). Both supervised and unsupervised methods have been active areas of research in the past decade, with network architectures such as U-Net (Ronneberger *et al.*, 2015 ▸; Siddique *et al.*, 2021 ▸), the mixed-scale dense (MSD) network (Pelt & Sethian, 2018 ▸), and variations of those, having much success with denoising and segmenting SRµCT images.

In the unsupervised learning space, Noise2Inverse (N2I) was first introduced by Hendriksen *et al.* (2020 ▸, 2021 ▸) as a deep learning method to denoise images without high-quality reference data. In bone research, this method is highly advantageous because high-quality reference data come at the cost of radiation damage in biological tissues. Instead of using a high-quality input, N2I splits a sinogram from SRµCT images to obtain several sub-reconstructions of lower quality, which are typically trained against one another. Since then, several variations and improvements have been suggested for this training method (Lagerwerf *et al.*, 2020 ▸; Wirtensohn *et al.*, 2023 ▸). Recently, Sparse2Inverse has been proposed as a self-supervised method that uses a loss function in the projection domain to reduce low-projection artefacts (Gruber *et al.*, 2024 ▸). Rotationally augmented N2I takes advantage of the equivariant property of the rotation transform to also aid in reducing low-projection artefacts (Xu & Perelli, 2024 ▸). While these networks have indeed shown great promise for application in the bone research field, direct application of unsupervised/self-supervised techniques to SRµCT data is currently limited due to the emerging developing state of these techniques.

Supervised methods of denoising and segmentation were recently used to quantify bone-crack growth mechanisms from low-dose *in situ* SRµCT imaging by Sieverts *et al.* (2022 ▸), which serves as the backbone for this work. While fractures were quantified, the volume and shape of lacunae in bovine bone were not assessed in our previous work. Microcracks and lacunae area were quantified in human trabecular bone during an *in situ* SRµCT compression test by Buccino *et al.* (2023 ▸), though individual lacunae volumes and mineralization were not compared with typical values. There is a need to test network performance, not only with network performance metrics but also with commonly used metrics from each research field. In this study, we seek to examine metrics that are relevant to bone disease or mechanical properties such as lacunar volume, aspect ratio and mineralization.

In this work, we address the problem of radiation dose to make *in situ* SRµCT imaging of bone possible while also assessing the performance of N2I through common bone quality measures such as lacunar volume and mineralization. Previously, we performed *in situ* SRµCT imaging on bovine bone in a saline solution bath with low-dose imaging parameters (Sieverts *et al.*, 2022 ▸). In the first experiment in the present work, we trained multiple networks using N2I on low-dose datasets at full, one-half, one-third, one-fourth and one-sixth simulated doses to determine the lowest dose that can be delivered while maintaining high image quality. Using simulated doses, we assessed the performance of each network with parameters commonly studied in bone SRµCT imaging. In a second experiment, N2I was trained and applied on larger datasets with more training data at full and one-third simulated doses to determine its applicability, benefits and limitations for larger datasets of noisy data.

## 2. Methods

### 2.1. Study design

This study aims to determine the viability of N2I on *in situ* SRµCT mechanical testing. To this aim, *in situ* data acquired from a previous study were used to perform two experiments. The first experiment involves training five neural networks using N2I to denoise low-dose SRµCT images. The five neural networks correspond to five simulated radiation doses that were used to determine the most feasible balance between radiation dose, image quality and microstructure quantification. The doses were full dose, one-half dose, one-third dose, one-fourth dose and one-sixth dose. Training during this experiment was performed on one SRµCT scan to quickly determine the most promising of the five simulated doses. After a promising simulated dose level was determined, a second experiment was performed to determine differences between a network trained on low-dose images and a network trained on full-dose images. For this second experiment, the two neural networks were trained using N2I on eight SRµCT scans and statistically compared by quantifying microstructural features.

### 2.2. Bone sample preparation

**Figure 1:**
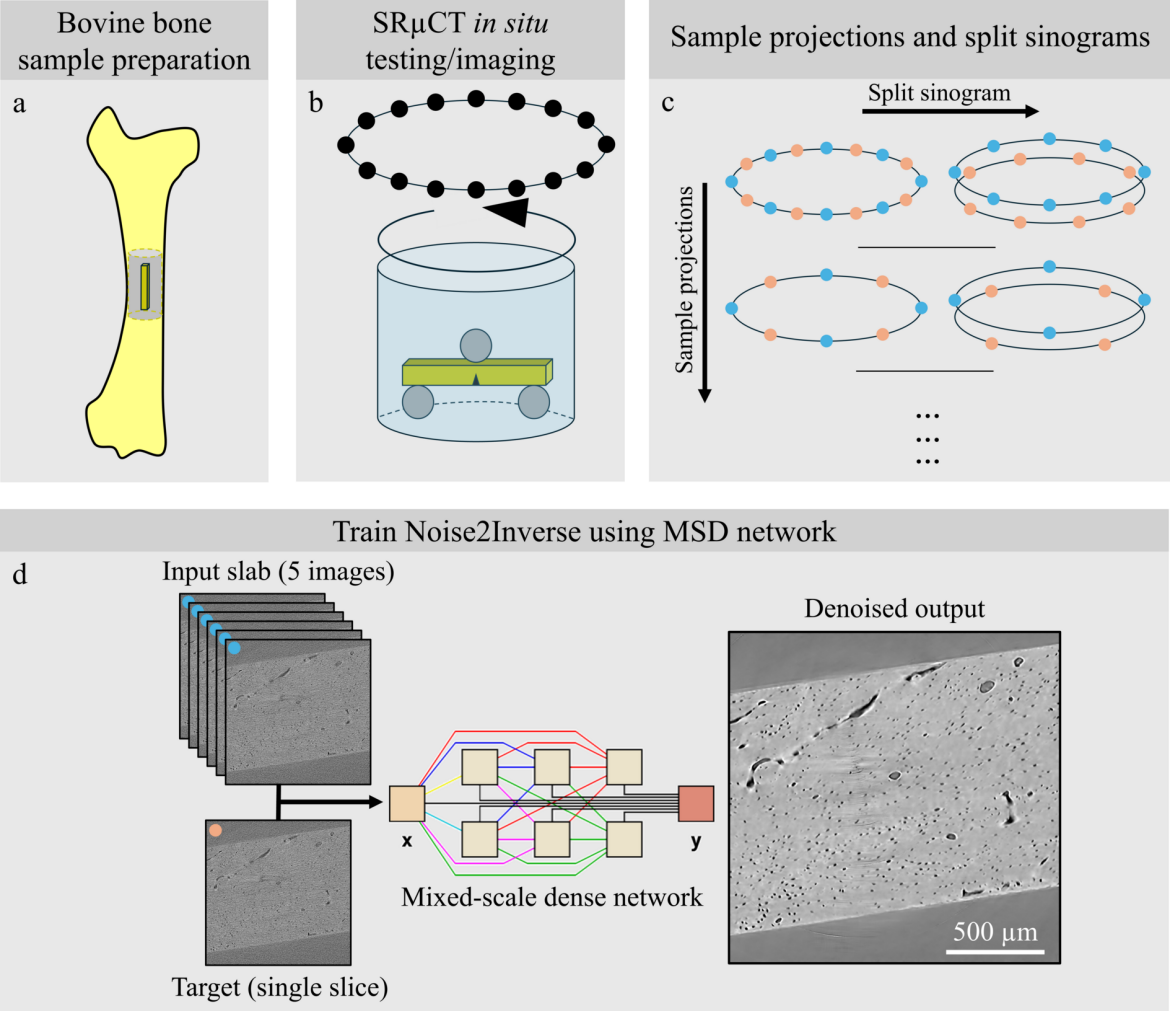
Experimental workflow for using N2I on *in situ* SRµCT tested samples. (*a*) Bone samples were extracted from the mid-diaphysis of a bovine femur. (*b*) These samples were subsequently notched and scanned in a hydrated *in situ* testing chamber where reference scans were obtained. (*c*) The sinogram was then split into *K* = 2 sub-reconstructions. The projections were also sampled to obtain varying dose simulations (Hendriksen *et al.*, 2020 ▸, 2021 ▸). (*d*) The images obtained through splitting and sampling of SRµCT data were used to train using N2I with the MSD network [portions of figure adapted from Pelt & Sethian (2018 ▸)].

Bone sample preparation and imaging were performed according to our previous study (Sieverts *et al.*, 2022 ▸). Cortical bone samples were processed from the mid-diaphysis of adult bovine femurs obtained from a local butcher shop. First, bone samples were cut longitudinally with a low-speed diamond saw. Subsequently, they were ground and polished under irrigation to dimensions of 1 mm × 2 mm × 10 mm. All bone samples were pre-notched with a custom-made razor micro-notcher under irrigation with a diamond suspension in preparation for fracture toughness testing [Fig. 1 ▸(*a*)].

### 2.3. Imaging of bovine bone samples at a synchrotron microtomography beamline

Following bone sample preparation, samples were scanned at the Advanced Light Source, beamline 8.3.2, at Lawrence Berkeley National Laboratory [Fig. 1 ▸(*b*)]. An X-ray energy of 24 keV with a 50 µm LuAG scintillator was used to obtain an image with a 3.4 mm field of view and 1.6 µm pixel size. A reference high-quality scan followed by low-quality scans were taken of the same sample with 3937 and 657 projections, respectively, and 100 ms exposure time at each step during incremental *in situ* imaging. For this work, the high-quality scan is sampled into low-dose simulated datasets. A detailed procedure for the *in situ* test protocol was outlined in a previous work (Sieverts *et al.*, 2022 ▸). Each bovine bone sample was imaged in an environmental chamber, submerged in a bath of phosphate-buffered saline (PBS) solution to simulate physiological conditions. Reconstruction of SRµCT data was performed using filtered back projection in Python open-source package *Tomopy* (Gürsoy *et al.*, 2014 ▸), and visualization was performed in *Dragonfly* (Comet Technologies, 2022.2). For visual comparison of some results, Paganin phase retrieval was performed (Paganin *et al.*, 2002 ▸; Mokso *et al.*, 2013 ▸). Furthermore, δ (1.1378 × 10^−6^) and β (4.8945 × 10^−9^) coefficients were determined using the Center for X-ray Optics Database with parameters corresponding to hydroxyapatite, and phase retrieval was performed using Python (Henke *et al.*, 1993 ▸; Sieverts *et al.*, 2024 ▸).

### 2.4. Dose-simulation datasets

To determine the performance of N2I in several scanning scenarios, dose simulations were created using the high-quality dataset, 3937 projections, from the *in situ* experiment. These projections were sampled to create sub-reconstructions at ratios of one-half, one-third, one-fourth and one-sixth of the original full dose, 3937 projections. With this method, each sub-reconstruction can still be compared with the reference full-dose image.

To determine practical radiation doses, the radiation dose imparted by each simulated scan was calculated using parameters of image acquisition and parameters of the beamline itself. Initially, the mass-attenuation coefficients for each medium the beam traveled through during the scan (water and bone) were obtained from the National Institute of Standards and Technology (NIST) database (Hubbell & Seltzer, 2004 ▸). The geometry of the testing chamber was used to estimate the thickness of each medium through which the beam had to pass to reach the region of interest for the sample at each given projection angle. These thickness values were used to estimate effective flux densities of between 25300 and 137000 photons s^−1^ µm^−2^, depending on the orientation of the testing setup and transmission of the materials passed through. While the beam traveled through a larger region within the bone, the cross section of the region of interest used for radiation-dose calculations was ∼3.28 mm^2^. A shutter was used during imaging to minimize the radiation dose delivered during the continuous rotation scan. Radiation-dose calculation equations were obtained from Barth *et al.* (2010 ▸) and are detailed in Section 2 of the supporting information. The number of projections, estimated dose and number of scans were calculated to ensure they remained below the recommended threshold of 35 kGy (Barth *et al.*, 2011 ▸), and are summarized in Table 1 ▸.

### 2.5. Denoising SRµCT images with Noise2Inverse

After the simulated-dose datasets were reconstructed, an MSD network architecture was used to train N2I with 100 layers (depth = 100) and 32-bit grayscale reconstructed SRµCT images as training data. A window of 1024 × 1024 pixels on 800 image slices was used during training for this first experiment. This same region was also used to quantify the mineralization and lacunae within the bone. The MSD network was trained using a learning rate of 10^−3^, an input slab size of 5, and a batch size of 12 based on a hyper-parameter grid search of several learning rates and batch sizes. A training–validation split of 80–20 was used in the first dose experiments, with the data itself used as the test set.

To create the sub-reconstructions, each sinogram is split into sub-sinograms by taking every *K*th angle, θ. The number of splits, *K*, is a hyper-parameter of this technique; for this study, *K* = 2 [Fig. 1 ▸(*c*)]. This *K* was chosen because the number of projections in the low-quality tomography data was 657. This relatively low amount of projections is not suitable for a higher number of splits, *K*, because although the theory of the N2I technique shows that uncorrelated noise can be removed from images, reconstruction artefacts from low projections would be present. For example, *K* = 3 splits in a scan with 657 projections would result in 219 projections per sub-sinogram, resulting in severe low-projection artefacts in the final reconstruction.

These sub-sinograms were then reconstructed using filtered back projection, resulting in *K* = 2 sub-reconstructions of our low-quality data. A typical N2I approach involves taking the mean of *K* − 1 sub-reconstructions and using the remaining sub-reconstruction as the target; however, with *K* = 2, one sub-reconstruction can simply be trained as the input with the other as the target. In the case of *K* > 2, the mean of the new combination of sub-reconstructions would need to be taken again to be used as the input to the network. After training, the MSD network is applied to all sub-reconstructions, *K*, and the network output is averaged to obtain the final output of the trained network [Fig. 1 ▸(*d*)].

In the first experiment, this process was performed on the full dose, one-half dose, one-third dose, one-fourth dose and one-sixth dose simulation datasets, resulting in network outputs from five different low-dose imaging scenarios. In the second experiment, networks were trained on a larger dataset for the full dose and one-third of simulated doses, as these possessed promise for application on *in situ* experiments. A total of eight samples were used, with 8072 images used for training and 890 images used for validation. The same process for creating the simulated-dose data for training and validation was employed from the first dose-simulation experiment. After the networks were trained, microstructural features were analyzed in all the samples and compared using a paired t-test to assess changes in quantification.

### 2.6. Quantification of lacunae and mineralization in bone

After the networks were trained and applied to the input image data, lacunae were segmented using a Yen threshold in *ImageJ* (Fiji) (Schindelin *et al.*, 2012 ▸). This technique was chosen to uniformly and accurately segment lacunae in each of the samples without introducing human bias in threshold selection. After segmenting the lacunae, the lacunae image masks were imported into the image-processing software *Dragonfly* (Comet Technologies, 2022.2) for quantification. A connected component analysis was performed using a 26-connected particle labeling scheme and only particles of volume 50–2000 µm^3^ were considered. This range was chosen based on other studies measuring lacunae in 3D (Carter *et al.*, 2013 ▸; Dong *et al.*, 2014 ▸). Mode lacunae volume and aspect-ratio data were exported for each simulated dose and analyzed in Python. In the second experiment, a paired t-test was used to compare the one-third- and full-dose datasets. Prior to performing the test, a Shapiro–Wilk test for normality and a Levene test for equal variance were performed on the data.

Mineralization in the bone tissue was calculated after a correction for absorption through various media. First, the density of the surrounding media (water/phosphate-buffered saline solution) and the top roller used for three-point bend testing (alumina) were calculated using the coefficient of attenuation for each material according to NIST (Hubbell & Seltzer, 2004 ▸). The values obtained from this conversion were compared with known densities of water and alumina. A linear conversion equation was subsequently derived from the calculated values and the known values and applied to the hydroxyapatite in bone tissue. This process was performed for each dose simulation to determine the final mineral density of bone tissue in each sample. Quantification of mode mineralization was performed in *ImageJ* (Fiji) and analysis was performed in Python. In the second experiment, a paired t-test was used to compare the one-third- and full-dose datasets. Prior to performing the test, a Shapiro–Wilk test for normality and a Levene test for equal variance were performed on the data.

## 3. Results

### 3.1. Comparison of one-sixth dose simulated data with equivalent *in situ* experimental data

To determine the difference between the dose-simulation datasets and an experimental dataset acquiring the same number of projections, a simulated one-sixth dose reconstruction was compared with the previously acquired low-quality images. Mean squared error (MSE), peak signal-to-noise ratio (PSNR) and structural similarity index (SSIM) were calculated, and are shown in Table 2 ▸. In the simulated dataset, MSE, PSNR and SSIM changed by −17.3%, 4.1% and 34.3%, respectively. Simulated datasets possess lower MSE and higher PSNR and SSIM as expected because they are directly derived from the high-quality data. In contrast, the experimental low-quality data are taken sequentially after the high-quality reference data, resulting in minor changes. While the simulated data of the one-sixth dose simulation outperform the experimental data, the metrics remain within reason for estimation of experimental data.

### 3.2. Network performance for simulated doses using Noise2Inverse

**Figure 2:**
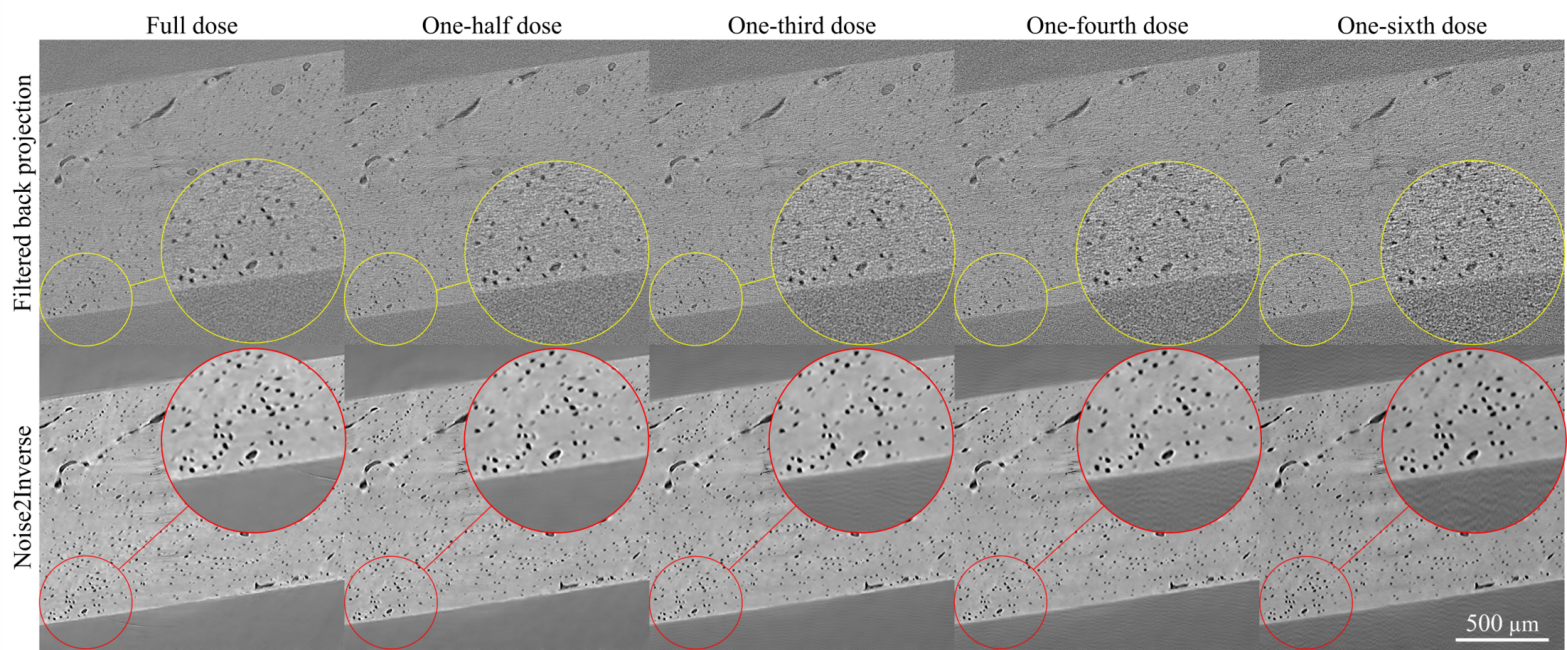
N2I results for full, one-half, one-third, one-fourth and one-sixth dose images are shown with their corresponding equivalent simulated-dose reconstruction. All N2I outputs (bottom row) are considerably denoised compared with the filtered back projection reconstructions (top row). Notable streak artefacts are found in the one-sixth dose image (wavy lines/ripples), where a lack of projection data induces artefacts in the background.

N2I reduced noise in all dose simulations (Fig. 2 ▸). Simulated-dose reconstructions, created with filtered back projection, are shown in the top row of Fig. 2 ▸ and the network output trained with N2I is shown in the bottom row. As the simulated dose decreases, the filtered back projection images become noisier. Additionally, at low simulated doses, streaking artefacts in the reconstruction are prominent in the background. After denoising with N2I on each dataset, the noise in each image is greatly reduced, as shown visually for every simulated dose. Although all simulated doses experience improved clarity, subtle changes in the shape of features are present and are exhibited when quantifying the lacunae and mineral content in the bone tissue.

### 3.3. Lacunae volume and aspect ratio distributions are shifted with lower simulated doses

**Figure 3:**
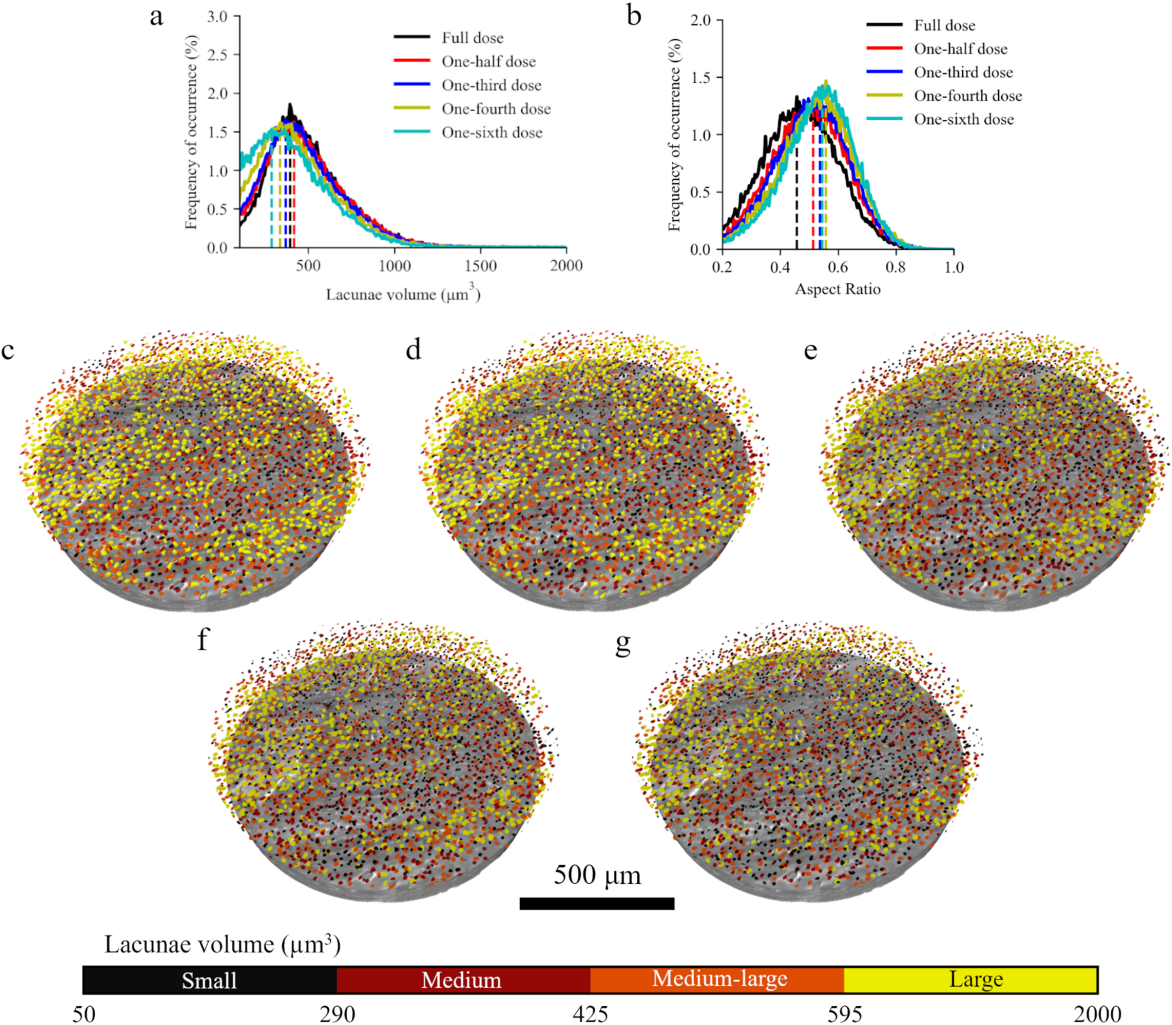
Shape and volume of lacunae from images denoised with N2I. (*a*) Lacunar volume was assessed for each of the networks, revealing that full-dose, half-dose and one-third dose experiments possessed similar lacunae volume distributions compared with the one-fourth and one-sixth dose simulated data. (*b*) Lacunae aspect ratio, the ratio of the minimum to the maximum eigenvectors of each lacunae, was compared for each of the simulated doses, revealing that lacunae tended to become more circle- or sphere-like as dose decreased compared with the more rod-like shape in the full-dose lacunae. (*c*)–(*g*) Representative 3D SRµCT images show the similarity between the full dose and one-third dose lacunae segmentations, whereas lacunae in the one-sixth dose segmentation may be larger and fewer.

Lacunae volume and aspect ratio are altered with dose reduction below one-third simulated doses (Fig. 3 ▸). We calculated a mode lacunar volume of 393 µm^3^ for the full-dose network, with one-half and one-third simulated doses exhibiting values of 417 µm^3^ and 368 µm^3^, respectively (6% change and −6% change).

The one-fourth and one-sixth simulated dose data underestimate the lacunar volume, with volumes of 336 µm^3^ and 287 µm^3^, respectively (−14% change and −27% change). Lacunae aspect ratios, or the ratio of the minimum to the maximum eigenvectors of each lacunae, for each subsequent dose simulation increased. This indicates rounder more spherical lacunae compared with the full-dose network results (0.46). Specifically, aspect ratios for the one-half, one-third, one-fourth and one-sixth simulated dose samples were 0.51, 0.54, 0.56 and 0.55, respectively (12.3%, 17.6%, 22.0% and 19.3% increase).

### 3.4. Mineralization distribution shifts to lower values with each simulated dose

**Figure 4:**
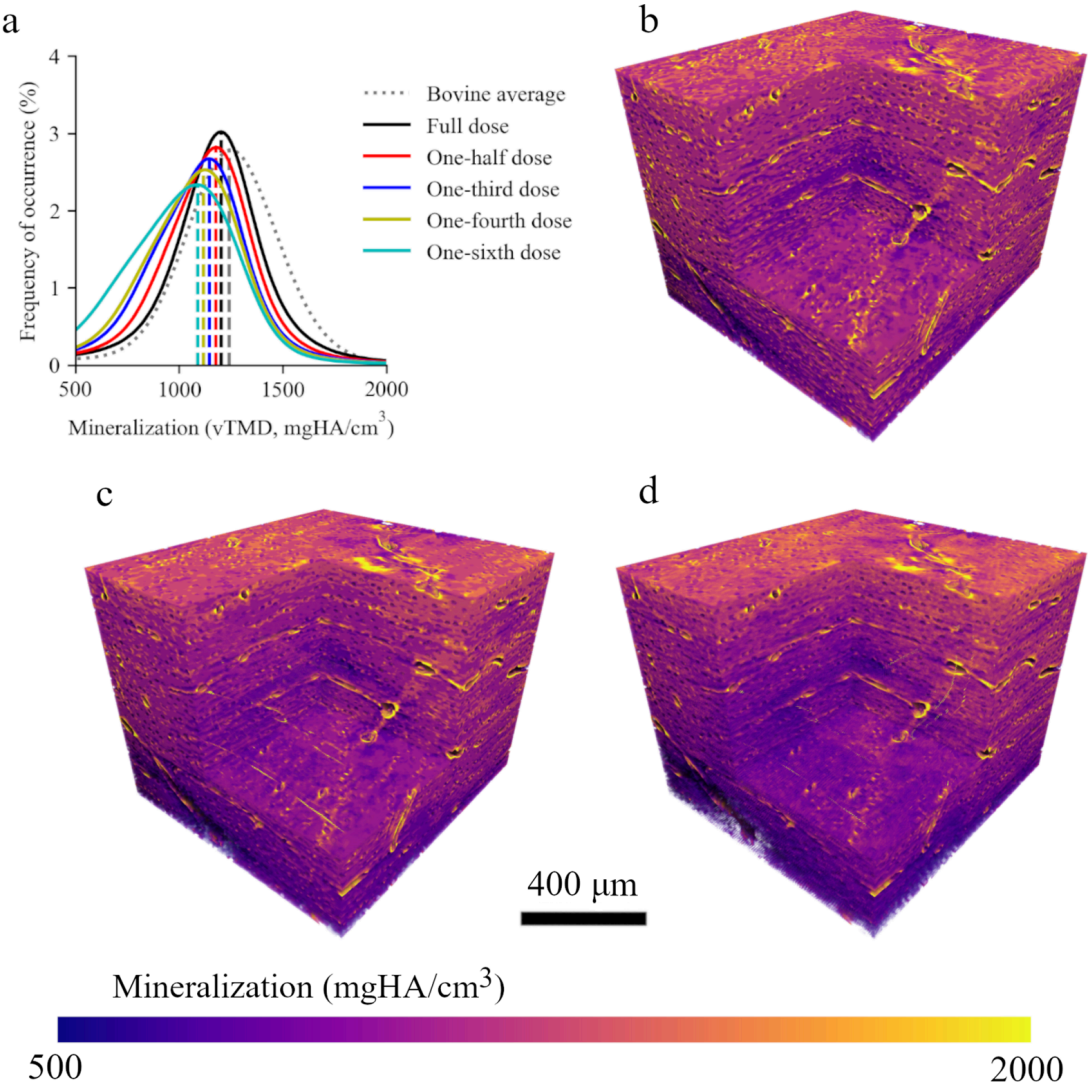
Mineralization of each simulated dose. (*a*) Mineralization distributions for each simulated dose and an averaged mineralization curve for several bovine bones. (*b*)–(*d*) Bone volumes in order of full, one-third and one-sixth dose simulations showing mineralization. As the simulated dose decreases, the values for mineralization shift to lower values.

Mineralization was also assessed for each of the simulated doses to determine possible changes induced by N2I (Fig. 4 ▸). Full-dose simulated results for mineralization (1202 mgHA cm^−3^, −2.0% change) were very similar to those typically observed in bovine bone (gray curve, 1226 mgHA cm^−3^). Each subsequent simulated dose reduced the mineralization calculated and increased the width of the distribution. Specifically, one-half, one-third, one-fourth and one-sixth dose samples possessed mineralization values of 1177 mgHA cm^−3^, 1146 mgHA cm^−3^, 1118 mgHA cm^−3^ and 1088 mgHA cm^−3^, corresponding to −4.1%, −6.6%, −8.8% and −11% changes.

### 3.5. Noise2Inverse allows segmentation of images where thresholding is not sufficient

**Figure 5:**
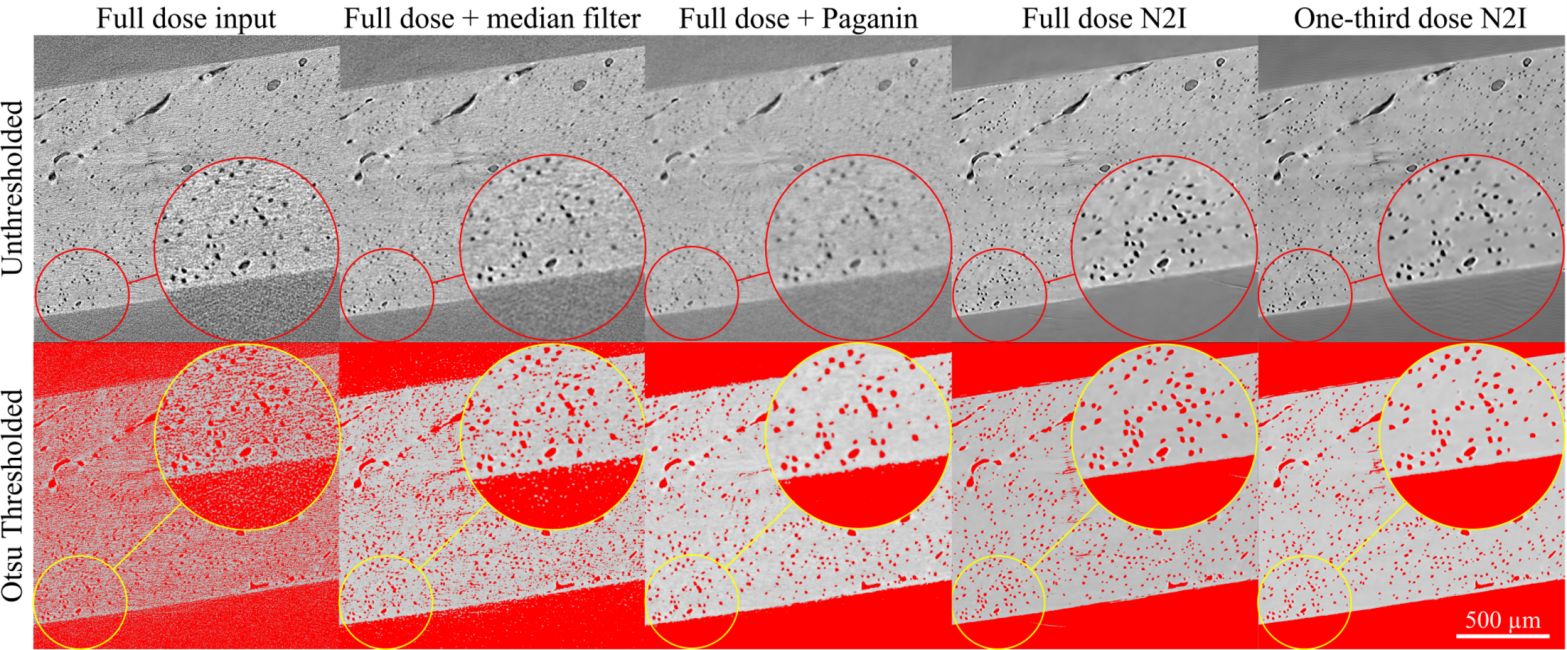
Input images, median-filter results, Paganin phase retrieval results and N2I results trained on eight samples for full-dose and one-third dose experiments (top row) are shown alongside an Otsu threshold (bottom row). In both the full-dose input image and the full-dose input image with a median filter applied, excess noise is captured, making segmentation of features using traditional thresholding techniques impossible. The full-dose image with Paganin phase retrieval was smoothed with features segmented using an Otsu threshold.

After performing the first dose experiment to determine the most promising doses for experimental use, the second experiment was performed where the full dose and one-third dose samples were trained on a larger dataset of eight SRµCT scans to assess statistical differences on the one-third dose simulation (Fig. 5 ▸). These datasets were also compared visually with a median filter as well as Paganin phase retrieval, popular methods of denoising SRµCT images (Sun & Neuvo, 1994 ▸). In Fig. 5 ▸, the top row indicates the image unthresholded, while the bottom row displays the same image with an Otsu threshold applied to demonstrate segmentability. The raw full-dose image was not segmentable with an Otsu threshold. The median filter applied to the raw data improved the Otsu segmentation; however, many small particles of noise are present in this image. Using Paganin phase retrieval on the full-dose image, Otsu thresholding was enabled with features clearly visible. Both the full dose and one-third dose N2I allow for segmentation of pores with a simple Otsu threshold. Notably, the one-third dose N2I output enabled similar performance to the full-dose images with Paganin phase retrieval and the full-dose N2I output.

### 3.6. Quantification of microstructure shows differences in dose simulations

**Figure 6:**
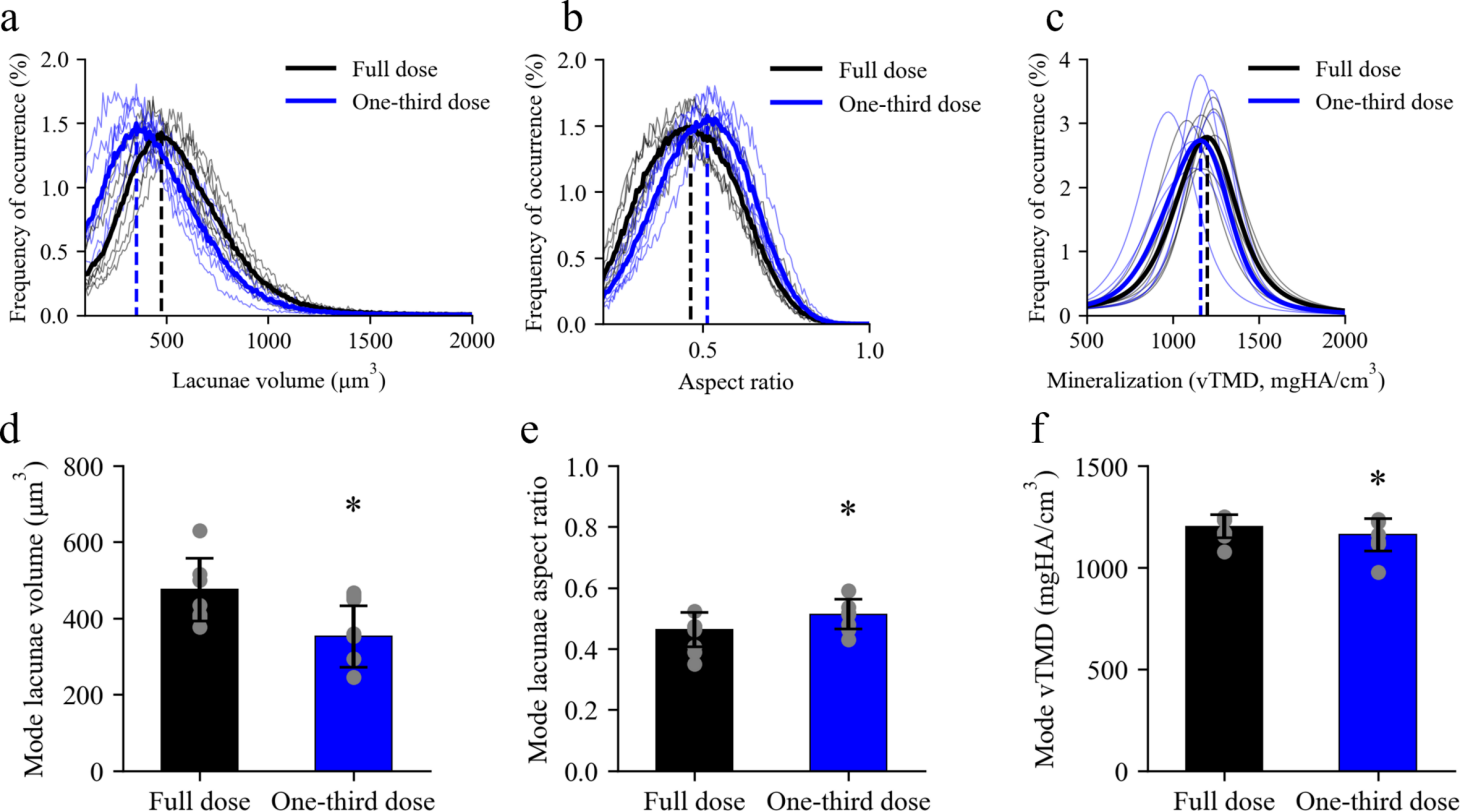
Quantification of microstructural features for all samples used in full-dose and one-third dose N2I training. (*a*) Lacunae volume distributions varied significantly from the full-dose to the one-third dose experiments. The one-third dose experiments measured lower-volume lacunae. (*b*) Lacunae aspect ratio increased in the one-third dose simulations compared with the full-dose simulations. (*c*) Mineralization significantly decreased in the one-third dose simulations compared with that of the full-dose experiments. (*d*)–(*f*) Lacunae-volume, aspect-ratio and mineralization mode values are significantly shifted in the one-third simulated-dose samples compared with the full-dose simulated data (*p* < 0.01 for all). Data are given as the mean ± standard deviation. All statistics were performed with a paired t-test in Python.

To assess the variance of the network results, each network was applied to eight samples and microstructural features were quantified. Histograms for lacunar volume, aspect ratio and mineralization are shown for all eight samples in Figs. 6 ▸(*a*)–6 ▸(*c*), with mean distributions highlighted. The mode of each distribution significantly changed between the full-dose experiment and the one-third dose experiment, with −26% change in lacunae volume, 10% change in lacunae aspect ratio and −3.4% change in mineralization [*p* < 0.01 for all, Figs. 6 ▸(*d*)–6 ▸(*f*)].

## 4. Discussion

In applications with time-resolved experiments where taking a high-quality reference scan is not feasible, N2I possesses high applicability due to its lack of reliance on high-quality training data. When using a supervised training approach, high-quality reference images can either be taken before or, preferably, after a test is performed; however, training data would be limited by the number of samples tested. Using N2I, training data scale with the number of sub-reconstructions, *K*, providing more examples for a network to train on. Performance of N2I may vary depending on whether the data are trained using reconstructed data or data in the projection domain, but this was not explored in the current work. In the projection domain, changes to some methods such as altering the loss function may be required, as shown by Gruber *et al.* (2024 ▸) with Sparse2Inverse. Additionally, the creation of *K* sub-reconstructions for each scanned sample increases the amount of training data available, and, in turn, increases the total data required. While not affecting data acquisition, this makes data management during training for generalized models a difficult task because many terabytes of data typically need to be managed with this technique.

While all images exhibited significant denoising, differences emerged when quantifying microstructural characteristics in each of the network outputs. In the first experiment, the distribution of lacunar volumes changed slightly in the outputs corresponding to full, half and one-third doses. In contrast, those from one-fourth and one-sixth doses displayed a noticeable shift towards lower volumes [Fig. 3 ▸(*a*)]. For the data trained on the single sample here, reducing the number of projections by one-third and applying N2I had a smaller effect on bone lacunae and mineralization measurements, showing promise. Based on Table 1 ▸, one-half and one-third dose (4–2.7 kGy) data in this study are well suited for *in situ* SRµCT experiments with multiple time steps required. However, all lacunae in the one-half to one-sixth dose experiments exhibited higher aspect ratios (more spherical than rod-like) compared with lacunae in the full-dose network. This was further investigated in the second experiment.

In the second experiment with the larger dataset, significant changes were observed in lacunar volume and aspect ratio in a paired t-test (Fig. 6 ▸). Thus, validation of measurements on data denoised using N2I in cases with noisy non-segmentable reference images is recommended. For example, lacunae measurements could be validated with a scan not taken under *in situ* imaging conditions and many projections if volumetric accuracy is desired. Accuracy of measurements is dependent on feature size as well, as lacunae are on the smallest end of features accurately quantifiable using the pixel size of 1.6 µm (Williams *et al.*, 2021 ▸). Based on work from the original N2I study (Hendriksen *et al.*, 2020 ▸) with an absolute ground truth, N2I is successful for denoising µCT images; however, in this work we see the effect of sampling projections from a reference dataset that is not an ideal ground truth. Additionally, the original work from Hendriksen *et al.* (2020 ▸) includes a mathematical proof that states that N2I converges to the unknown noise-free reconstruction when given an infinite number of training examples. In practice, the number of training examples is finite, but this principle suggests that when the amount of training data is sufficiently large (*e.g.* by combining multiple datasets), distortions of the volume and shape of small features may be mitigated.

To further examine the causes of the small feature distortions, N2I was employed on a separate bovine bone scan with 1969 projections. This scan was sampled to its half dose (984 projections), where *K* = 2 sub-reconstructions were then taken. The sample was mounted vertically while the beam passed through no other medium than air (Fig. 1 of the supporting information). A 5% change in lacunae volume and a 10% change in lacunae aspect ratio were observed in the half-dose N2I compared with the ground truth. This change was statistically significant, showing that increases to lacunae volume and shape certainly occur as a result of sampling scans with too few projections. The projections used in this test correspond to the same number of projections as the one-fourth dose scan. Although the data had low noise with 1969 projections, the simulated half dose may not have possessed enough projection data when sub-sampled for accurate results. This shift is another important consideration when small-feature quantification is desired. Based on the work here, lacunae quantification in low-dose datasets is challenging for a pixel size of 1.6 µm.

Despite the implementation of a gray-value correction in each simulated-dose output, a trend towards decreased mineralization is evident with decreasing simulated dose. Low-projection artefacts, manifested as streaking, are observed in simulated doses at one-third and below, particularly noticeable within the PBS background of the image. Although predominantly observed in the background, these streaks are also discernible within the bone tissue, potentially influencing mineralization measurements, given their linear relationship with gray values in the image. Given that several bone pathologies are characterized by diminished mineralization levels, caution should be exercised when extrapolating implications for bone diseases from outputs generated by low-dose networks with noisy reference data. Despite the small 3% change in mineralization, this statistically significant change indicates that drastic measures of decreasing radiation dose introduce error in calculated mineralization when reference data are noisy.

Through investigation of lacunae, mineralization, and by testing N2I on a conventional SRµCT scan, we have determined that there are two sources of feature distortion: low number of projections in sub-reconstructions and high noise in the reference images. We believe high noise in the reference images stems from two main factors of our *in situ* mechanical test. Firstly, high X-ray attenuation; imaging through the PBS solution attenuates the X-rays, reducing the signal (Li & Tang, 2019 ▸). Secondly, the sample is larger than the field-of-view and has complex structure, creating distortions during reconstruction. Combined, these factors are likely responsible for the high noise in the reference scans and, in turn, the feature distortion of lacunae. Although some of these factors, such as the size of the sample, may not be flexible to change for each *in situ* SRµCT experiment, future *in situ* experiments can optimize data acquisition with these factors in consideration.

Based on the results from each experiment performed in this work, we can make four recommendations when using N2I:

(1) Performing dose calculations prior to testing as well as estimating the number of scans necessary for each experiment/sample is imperative. During this step, planning low-dose scan parameters with a beamline scientist is useful because acquired data from a low-dose scan will be noisy. This noise makes real-time reconstruction of the data an unclear method of ensuring quality because a neural network will not be ready for denoising data immediately as it is acquired. In turn, the extent to which the data are denoised for analysis is not known directly at the time of imaging.

(2) Imaging with parameters to achieve 4–2.7 kGy (one-third to one-half dose in this work) per scan is preferable from an experimental perspective, as numerous scans can be obtained without exceeding the 35 kGy recommended limit (Barth *et al.*, 2010 ▸). This also reiterates the importance of performing dose calculations prior to testing, as imaging parameters and X-ray flux can vary from beamline to beamline.

(3) Based on the use of N2I here, the number of projections acquired during data acquisition should be maximized for a given dose to reduce low-projection artefacts when the sinogram is split. To achieve this, optimum scanning parameters for N2I may require more projections and less exposure time than general low-dose tomography parameters.

(4) For accurate microstructural features, attenuation of the X-ray beam must be mitigated. This can be achieved by removing attenuating media, such as PBS, from the *in situ* test setup prior to imaging. Some pivotal factors such as sample geometry and orientation relative to the beam may introduce noise and be restricted by the experimental setup.

With these four considerations, N2I can effectively be applied to low-dose *in situ* synchrotron experiments.

## 5. Conclusions

Self-supervised methods for denoising have merit in dose-sensitive *in situ* SRµCT experiments. In particular, N2I shows promise for application to data that are obtained without a high-quality reference dataset, especially for data that cannot to be thresholded. The results here show comparable mineralization and lacunae morphology for self-supervised methods for simulated doses in the one-third to one-half dose range in this study. When training with more samples, significant changes in lacunae volume, aspect ratio and mineralization were observed. This shows that while self-supervised methods of deep learning show promise in the field of bone imaging, lack of projection data, sample geometry and experimental setup must be considered for small features and gray values to be representative of the ground truth. To prevent these feature distortions, optimizing scanning parameters specifically for N2I by increasing projections and decreasing exposure time may help to alleviate or eliminate these effects.

## Supplementary Material

Supporting information. DOI: 10.1107/S1600577525001833/vl5030sup1.pdf

## Figures and Tables

**Table 1:** Simulated radiation doses and number of scans until radiation damage

Simulated dose	Full	One-half	One-third	One-fourth	One-sixth
Projections	3937	1969	1313	985	657
Dose (kGy)	8.0	4.0	2.7	2.0	1.3
Full scans before radiation damage	4	8	13	17	26

**Table 2:** Simulated dataset and experimental dataset comparison

Dataset	One-sixth experimental	One-sixth simulated
MSE	0.00225	0.00186
PSNR	26.87	27.96
SSIM	0.35	0.47
