# Supplementary material for: Enhancing synchrotron radiation micro-CT images using deep learning: an application of Noise2Inverse on bone imaging
Source: J Synchrotron Radiat. 2025 Apr 1;32(Pt 3):690–9. doi: 10.1107/S1600577525001833 (PMC12067336; doi:10.1107/S1600577525001833)
Supplement: Supplementary file 1 [file s-32-00690-sup1.pdf]

JOURNAL OF SYNCHROTRON RADIATION

Volume 32 (2025)

Supporting information for article:

Enhancing synchrotron radiation micro-CT images using deep learning: an application of Noise2Inverse on bone imaging

Yoshihiro Obata, Dilworth Y. Parkinson, Daniël M. Pelt and Claire Acevedo

Supplemental Information: Enhancing synchrotron radiation micro-CT images using deep learning: an application of Noise2Inverse on bone imaging

Noise2Inverse on typical-dose SRµCT scans

To determine whether attenuation through media outside of the scanning window was the primary cause of feature distortion in SRµCT scans, N2I was applied to samples taken with a typical x-ray dose. These samples were scanned with 1969 projections and a beam energy of 18 keV. Images were reconstructed with filtered back projection with a pixel size of 1.6 µm. To assess the performance of N2I on this dataset, a simulated half dose (985 projections) was used, with $K = 2$ sub-reconstructions. The same network architechure from the main text was used for training. Data from eight samples was used during training and for bone feature quantification.

Distributions of lacunae volume and aspect ratio shifted to larger values shifted to larger values in the one-half dose with N2I. Specifically, mode lacunae volumes with filtered back projection were 364 μm^3, while mode lacunae volumes with N2I were 383 μm^3 (5% increase, Supplemental Fig. 1a, c). Mode aspect ratios with filtered back projection were 0.31, while mode aspect ratios with N2I were 0.34 (10% increase, Supplemental Fig. 1b, d). Visual comparisons of features are shown in Supplemental Fig. 1e, f.

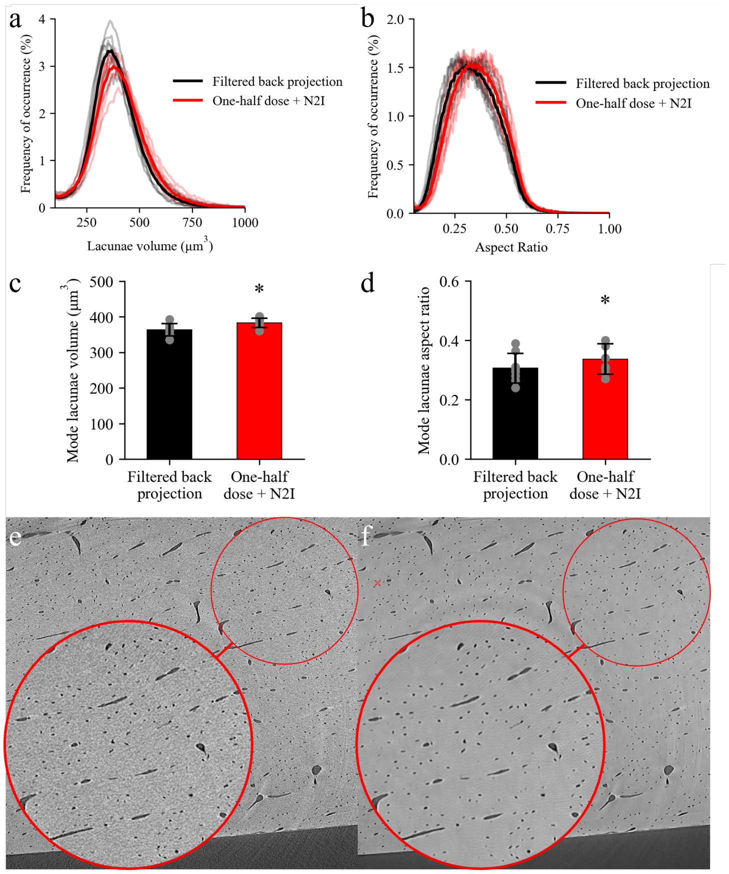

Figure 1: Lacunae aspect ratio and volume for bovine bone scanned in air mounted vertically with and without N2I. (a) Lacunae volume for bovine bones scanned with 1969 projections are plotted (black) compared to a trained Noise2Inverse network output (red) with one-half simulated dose, showing a 5% increase in mode lacunar volume. (b) Lacunae aspect ratio is plotted in a similar manner, showing a 10% increase in mode aspect ratio in the N2I data. (c-d) The modes of lacunae volume and lacunae aspect ratio are shown in bar plots, with bars and errorbars representing the mean and standard deviation of the mode values. Significant changes were found in both parameters when compared using a paired t-test. (e) Visual results of the high-quality filtered back projection data and the (f) one-half dose trained N2I are shown.

Dose Calculations

To begin, the radiation flux density, ψ, is calculated using the flux, ϕ, and area of the beam at the sample, z.

$$\psi = \frac{\phi}{z} \tag{1}$$

The effective flux density, $\psi_{effective}$, is then calculated depending on the transmission of x-rays traveling through attenuating media to reach the sample. Transmission is a function of the thickness of material, l, the mass attenuation coefficient, α, and the density of the material, ρ.

$$T = \frac{I}{I_0} = e^{-\alpha\rho l} \tag{2}$$

$$\psi_{effective} = \psi \times T_{water} \times T_{bone} \tag{3}$$

The thickness of water and bone the beam must travel through is estimated using the geometry of the *in situ* mechanical testing chamber and the geometry of the sample itself and is shown in Figure 2 below.

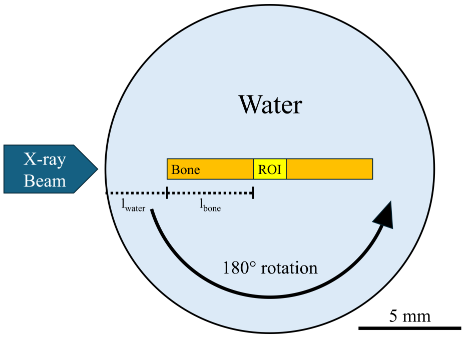

Figure 2: A top down schematic of the testing chamber. Thicknesses of water and bone are shown for a projection angle of 0 here. As the stage rotates, the thicknesses of water and bone the beam must travel through to reach the ROI change.

The effective flux density is then converted to an energy density, E_ρ, using the beam energy, E.

$$E_\rho = \psi_{effective} \times 1.6 \times 10^{-19} J/eV \times E \tag{4}$$

Following this, the absorption, A, of x-rays through the region of interest in the scan using Equation 2 and the following relationship.

$$A = 1 - T \tag{5}$$

The dose rate, $\dot{d}$ is then calculated using A, E_ρ, cross-sectional area of the region of interest being irradiated, a, and the mass of bone in the region of interest absorbing the radiation, M.

$$\dot{d} = \frac{AE_\rho a}{M} \tag{6}$$

The total radiation dose imparted on the sample is then calculated using the dose rate and total exposure time, t. It should be noted here that time will depend on whether a continuous scan was performed or whether a shutter was used during imaging to limit total exposure time.

$$Total\ irradiated\ dose = \dot{d} \times t \tag{7}$$
